# ﻿Comment and integration to Andreone et al. 2022 “Reconnecting research and natural history museums in Italy and the need of a national collection biorepository”

**DOI:** 10.3897/zookeys.1137.96414

**Published:** 2022-12-22

**Authors:** Marco Benvenuti, Fausto Barbagli, Francesca Maggiore

**Affiliations:** 1 SMA Museum System of the University of Florence, Via G. La Pira 4, 50121 Firenze, Italy SMA Museum System of the University of Florence Firenze Italy; 2 ANMS National Association of Scientific Museums, Via G. La Pira 4, 50121 Firenze, Italy ANMS National Association of Scientific Museums Firenze Italy; 3 CNR -ISMAR National Research Council - Institute of Marine Sciences, Arsenale - Tesa 104, Castello 2737/F, 30122 Venezia, Italy CNR -ISMAR National Research Council - Institute of Marine Sciences Venezia Italy

**Keywords:** Data digitisation, DiSSCo, FAIR, natural history museum collections

## Abstract

In Andreone et al. (2022), the authors described a background about the Italian Natural History Museums (NHMs) situation, highlighting difficulties regarding the coordination among institutes due to the fragmented landscape, from the past until today. They suggested how having a national institute, the Future National Biodiversity Centre (FCNB), woul d represent the best solution to the problem. Our vision regarding the lack of a national natural history museum in Italy does not coincide with that of the authors, but we do not consider clarifying this aspect in the present letter. On the other hand, since the authors reported how “the present fragmentation of museums and associated collections does not allow for an effective participation of Italy to global models of aggregated natural history databases (such as the VertNet, iDigBio, GBIF)”, we believe it is necessary to address the issue linked to the digital sharing of Italian collections. We present more clarifications about Italy’s commitment to the digitisation and sharing of NH collections data through the DiSSCo RI “Distributed System of Scientific Collections Research Infrastructure” of which Italy is one of the 23 European partner countries since 2018.

Dear Editor,

In the commentary “Reconnecting research and natural history museums in Italy and the need of a national collection biorepository” (Andreone et al. 2022) the authors described a broad background concerning the Italian Natural History Museums (hereafter NHMs) situation, providing an overview on the several difficulties about coordination among the different institutes. Our vision regarding the lack of a national natural history museum in Italy does not coincide with that of the authors, but we do not consider this the right place to propose an alternative view. On the other hand, we believe it is necessary to address the issue linked to the digital sharing of Italian collections. A proposal to establish a National Biodiversity Centre (Centro Nazionale per la Biodiversità, CNB) as a reference point for all the Italian NHMs has more recently been concretised within the Italian PNRR (National Recovery and Resilience Plan). There is an urgent need to improve digital tools and the interoperability among digital infrastructures and data management systems. This has been further put in evidence after the COVID-19 pandemic and one of the primary purposes of the PNRR is to improve the digitisation in any working field. Thus, it perfectly suits with the digitisation of museum collections, offering an incredible opportunity to finalise what has not been possible to do until now.

It is true that much work still needs to be done in Italy in order to digitise the Natural History Collections (hereafter NHCs) and to share the richness of the information embedded there (in both the biological and geo-mineralogical field) worldwide. Nevertheless, by only focusing on the national level, the authors seem unaware that a strong strategy to facilitate and improve NHCs interoperability already exists at the international (European) level. DiSSCo RI (Distributed System of Scientific Collections Research Infrastructure; https://www.dissco.eu/), indeed, aims to digitally unify all European NHCs thanks to shared access and curation policies and practices, ensuring that all the data and metadata are linked to a digital twin of the physical specimens (the so-called “Digital specimens”) and accomplish the FAIR (Findable, Accessible, Interoperable and Reusable) principles. DiSSCo is currently still in the preparatory phase, but some services are already developed and a very large scientific community (including Italian institutions) has been fully involved in this project for several years, tightly connected with a broader international panel of similar initiatives (GBIF, iDigBio, Atlas of Living Australia).

It is essential to underline that Italy is one of the 23 DiSSCo partner countries and has been taking part to this process since 2018 through a DiSSCo’s Italian consortium, led by the Florence NHM as National Node and also including eight other institutions representing the whole Italian NHCs community: CNR (National Research Council), ANMS (National Association of Scientific Museums), National Academy of Sciences, National Academy of Entomology, Italian Society of Biogeography, Italian Paleontological Society, Italian Geological Society, and the Italian Botanical Society (https://www.dissco.eu/it/). Moreover, Italy is represented in the CETAF (Consortium of European Taxonomic Facilities) by the Florence and Genoa NHMs since 2002, recently joined by the Pisa Herbarium and Botanical Garden (Bartolozzi 2013; Innocenti et al. 2017).

This is the first concrete step toward the alignment of Italian NHMs with the FAIR principles, which will make even the natural science data in our country shareable beyond the actual physical position, management, and constraints of the specimens themselves.

In this wider overview, the CNB (National Biodiversity Centre) would represent a node for communication and coordination at national level, connecting the Italian scientific community and the NHMs holding biological collections with DiSSCo, working in synergy to achieve a common final goal.

## References

[B1] Andreone F Boero F Bologna MA Carpaneto GM Castiglia R Gippoliti S Massa B Minelli A (2022) Reconnecting research and natural history museums in Italy and the need of a national collection biorepository. ZooKeys 1104: 55–68. 10.3897/zookeys.1104.79823 PMC9848790 36761931

[B2] Bartolozzi L (2013) I musei naturalistici italiani nel contesto delle iniziative internazionali sulla biodiversità. Museologia Scientifica, Memorie 9: 17–20.

[B3] Innocenti G Cianfanelli S Corti C Nistri A Nocita A Vanni S (2017) Researches on biodiversity at the Natural History Museum, Florence University. BioSyst.EU 2017, Abstract Vol. 9, 1–103. https://iapt-taxon.org/files/2017_BioSysteEu_Abstracts.pdf

